# Force distribution is more important than its intensity!

**DOI:** 10.1590/2176-9451.19.1.005-007.oin

**Published:** 2014

**Authors:** Alberto Consolaro

**Affiliations:** 1 Head Professor, FOB-USP. Professor of the Post-graduation Course, FORP-USP.

**Keywords:** Orthodontic forces, Induced tooth movement, Root resorption

## Abstract

A common question about root resorption is raised in orthodontic practice: What is more important, the intensity of force or its distribution along the root, periodontal and alveolar structures? Diffuse distribution of forces applied to periodontal tissues during tooth movement tends not to promote neither extensive areas of cell matrix hyalinization nor significant death of cementoblasts that lead to root resorption. However, focal distribution or concentration of forces within a restricted area - as it occurs in tipping movements, even with forces of lower intensity - tend to induce extensive areas of hyalinization and focal death of cementoblasts, which is commonly associated with root resorption. In tipping movements, the apical regions tend to concentrate more forces in addition to wounding the cementoblasts due to the smaller dimension of their root structure as well as their cone shape. For this reason, there is an increase in root resorption. In the cervical region, on the other hand, the large area resulting from a large diameter and bone crown deflection tends to reduce the effects of forces, even when they are more concentrated, thus rarely inducing death of cementoblasts and root resorption.

## Abstract (pt)

Um questionamento comum sobre as reabsorções radiculares na prática ortodôntica: "O que é mais importante? A intensidade das forças aplicadas ou sua distribuição ao longo das estruturas radiculares, periodontais e alveolares?" A distribuição difusa das forças aplicadas sobre os tecidos periodontais durante o movimento dentário de corpo tende a não promover extensas áreas de hialinização da matriz extracelular, nem morte significativa de cementoblastos que levariam à reabsorção radicular. Porém, a distribuição focal ou concentração de forças - como nas inclinações, mesmo nas de menor intensidade - em uma área restrita tende a induzir áreas extensas de hialinização e morte focal de cementoblastos, associando-se mais comumente à reabsorção radicular. Nos movimentos de inclinação, as áreas apicais, por sua menor dimensão da estrutura radicular e sua forma cônica, tendem a concentrar mais ainda as forças e lesar cementoblastos, aumentando a frequência das reabsorções radiculares. Na região cervical, a maior área decorrente do maior diâmetro e a deflexão óssea da crista óssea tendem a reduzir os efeitos das forças, mesmo quando mais concentradas, muito raramente induzindo a morte de cementoblastos e reabsorções radiculares.

The periodontal ligament is on average 0.25 mm (250 µm) thick, based on measurements that vary from 0.2 to 0.4 mm (200 to 400 µm). It uniformly comprises a membrane of highly specialized fibrous connective tissue of which total volume is occupied in 50% by blood vessels among which venules and capillaries are predominant. These vessels have the thinnest walls and can be easily constricted, which causes quick changes in periodontal ligament blood flow, even when lower forces are applied.

With regard to the forces applied during orthodontic movement, they are usually of low magnitude, even when, in the clinical practice, they are referred to as heavy forces: orthodontic forces cannot be compared to those of occlusal trauma or dental trauma. In order to move the teeth, it is necessary to excite, stimulate or stress the periodontal ligament cells so as to increase the local production of mediators that are usually released for intercellular communication. Locally increased mediators speed up metabolism and resorptive activity in the periodontal surface of the bundle bone, also known as the alveolar bone or primary and embryonic bone. The increased activity in the area where the ligament is constricted ends up directing tooth movement according to the objective of the clinical force application.^1^

In order to attain cellular stress, the force applied to the delicate tissue membrane known as periodontal ligament must be of very low magnitude due to the fact that its dimensions are diminutive with many easily constricted blood vessels. Stress can be caused by restricting the volume of blood by means of reducing vessel diameter, or by mechanical deformation of the cell cytoskeleton. Both ways - metabolic or mechanical - mediator liberation synergistically increase in the areas where the forces applied to the periodontal ligament during orthodontic movement act.

As for specialization of the periodontal ligament, it may be considered under many aspects,^1^ among which are the functions of:

1. Cementoblasts that produce the cementum that is necessary for structural and organizational integration of periodontal collagenous fibers with the tooth root.
2. Fibroblasts that orderly produce collagenous fiber structures distributed in such a way that ease and harmonize the forces applied during the function of the tooth, preventing it from touching and being constricted against the alveolar bone in a way that is too harmful for other periodontal structures, such as vessels and nerves.
3. Osteoblasts that produce the primary or bundle bone, an alveolar plaster responsible for speeding up the different renovations of the secondary or adult bone that comprises most of the human skeleton. This quick renovation is required by the continued use of tooth and ligament functioning as a joint with the alveolar bone.
4. Epithelial rests of Malassez, clusters of cells uniformly and randomly distributed among the periodontal components that constantly release the epidermal growth factor (EGF).^1^ This mediator continually keeps resorptive activity of the bone in the periodontal surface of the alveolar process, thus preserving the periodontal space, avoiding alveolodental ankylosis and its most feared consequence: Tooth resorption by replacement.

## INTRUSIVE FORCES

Intrusive forces applied to the teeth should be perpendicular to the bottom of the alveolus. Forces that are perpendicular to the bottom of the alveolus are efficiently softened and distributed along the alveolar process by means of the different forms of organization of the collagenous fiber structures in the periodontal ligament.

The periodontal ligament is specifically structured and organized so as to absorb the masticatory forces that tend to be intrusive or perpendicular to the bottom of the alveolar bone.

Intrusive forces are not aggressive to the periodontal tissues during masticatory function. In orthodontic practice, this type of force is not applied to any of the therapeutic procedures used.

Differently from the concept of intrusive forces are the techniques or protocols generally known as intrusive mechanics. This technique aims at producing an intrusive effect on the tooth in the alveolar bone: A real effect that is esthetically and functionally appropriate. Nevertheless, this effect can be produced by tipping movements, taking the position of the tooth in the alveolus, its tipping along the axis and the result of the applied forces into consideration in order to produce the intrusive effect. The applied forces are not at 90 degrees in relation to the bottom of the alveolus. In fact, it is not a pure intrusive force, but forces that lead to a real tipping movement and end up producing an intrusive effect, a real intrusion of the tooth in the alveolus, which is not a result of pure intrusive forces.

## BODILY TOOTH MOVEMENT

Bodily tooth movement in the alveolus resulting from forces applied to the crown are considered unreal, given that the entire root would hardly act uniformly, homogeneously and with the same force over the periodontal ligament in the entire surface of the alveolar bone where the force is applied.

Even though it is considered unreal, bodily tooth movement ends up distributing the forces orthodontically applied to the teeth in a more diffuse and less concentrated manner. In other words, the forces applied to the teeth in order to produce a bodily movement are better distributed in the periodontal and alveolar tissues. Constriction of periodontal vessels and, as a result, blood flow restriction as well as deformation of cytoskeletons are eased while the concentration of mediators is modulated in order to prevent the risk of cellular death and extracellular matrix hyalinization.

## TIPPING FORCES

The forces intentionally applied to produce tipping movements tend to be concentrated within a smaller periodontal area. For this reason, the risks of collapsed blood vessels, cellular death and extracellular matrix hyalinization are much higher. The tooth root has the cementoblasts as protectors of its structural integrity. Without receptors for the bone resorption mediators in their membrane, the cementoblasts are subject to tissue phenomena observed in tooth movement. When the cementoblasts eventually die due to forces that not only restrict the vessels that feed them, but also lethally deform them, there will be root resorption with denudation and exposure of the tooth surface facing the periodontal ligament.

The orthodontic forces tend to concentrate in the tooth apices due to their cone-shaped configuration, even when the objective is to produce bodily movement. In other words, when the forces act within a limited area, they tend to concentrate and increase the possibility of eliminating cementoblasts, thus increasing the frequency of root resorption in this region.

## FORCE DISTRIBUTION AND INTENSITY IN ROOT RESORPTION

With regard to orthodontically applied forces, the clinician must focus more on their distribution than on their intensity, especially with regard to induction of root resorption. If bodily movements are considered as a priority, even though it is known that they cannot be fully produced because there will always be some degree of tipping, the forces will be better distributed than when tipping movements are produced first.

Tipping movements tend to concentrate forces in the apical and cervical regions, in which case extracellular matrix hyalinization areas are established and some or many cementoblasts die. Tipping movements are more associated with apical root resorption.

The conical shape of the apical third favors greater concentration of forces in this region, with death of cementoblasts happening more often than in the cervical third. Larger diameter and area of the cervical third as well as the greater capacity of deflection of the bone crest ease the effects of forces concentrated in tipping movements. In the apical third, in tipping forces, there is no bone deflection and the root structure is narrower and thinner. In this region, the risks of collapsed blood vessels are higher than in the cervical region. These are the reasons why root resorptions in orthodontic practice are more common in the apical third and rarer in the cervical third.

When the main factor concerning the pathophysiology of resorptions is questioned, whether it is force intensity or distribution, it should be highlighted that distribution plays a more important role. Light, but concentrated forces promote resorption; while intense, but well distributed forces do not damage the periodontal structures so far as to cause death of cementoblasts, the initial phenomenon of root resorption.

## FINAL CONSIDERATIONS

Diffuse distribution of forces applied to periodontal tissues during orthodontic movement tends not to promote either extensive areas of cell matrix hyalinization or significant death of cementoblasts that lead to root resorption. However, focal distribution within a restricted area, even in cases of lower intensity forces, tends to induce extensive areas of hyalinization and focal death of cementoblasts, which is commonly associated with root resorption.

The apical regions tend to concentrate even more forces and wound the cementoblasts due to the smaller dimension of their root structure as well as their cone shape. For this reason, they are frequently associated with root resorption. On the other hand, in the cervical region, the large area resulting from large diameter and bone crown deflection tend to reduce the effects of forces, even when they are more concentrated, thus rarely inducing death of cementoblasts and consequently root resorption.

Clinical and image studies comparing techniques and shape of root and apex, as well as the different types of movements and forces, could support in an applied and direct manner the thoughts and insights on "orthodontic force intensity and distribution and its effects on root and periodontal structures: A comparative study according to different therapeutic protocols."

## References

[1] Consolaro A (2012). Reabsorções dentárias nas especialidades clínicas.

